# The clinical efficacy and mechanism of Rhodiola rosea in treating rheumatoid arthritis in high-altitude areas by regulating the HIF-1α signaling pathway

**DOI:** 10.3389/fmed.2026.1810329

**Published:** 2026-08-07

**Authors:** Qin Li, Guangzhao Zhu, Wenguang Zhang, Weihai Song, Zhiyi Zhuang

**Affiliations:** Department of Rheumatology and Immunology, Qinghai Provincial Hospital of Traditional Chinese Medicine, Xining, Qinghai, China

**Keywords:** HIF-1α signaling pathway, inflammatory cytokines, MMP-3, rheumatoid arthritis, Rhodiola

## Abstract

**Objective:**

To evaluate the clinical efficacy of Rhodiola rosea in high-altitude rheumatoid arthritis (RA) patients and to investigate, *in vitro*, whether its anti-inflammatory mechanism is mediated by the HIF-1α signaling pathway.

**Methods:**

This study adopted a retrospective analysis. Patients with RA in high-altitude areas were divided into the Rhodiola rosea treatment group (74 cases) and the conventional treatment group (86 cases). Baseline data, disease activity score (DAS28), inflammatory factors (CRP, ESR, TNF-*α*, and IL-6), serum HIF-1α levels, imaging data, and adverse reaction monitoring data were collected to evaluate the clinical efficacy and safety of Rhodiola rosea. Human synovial cells (HFLS-RA) were used for *in vitro* experiments to establish a hypoxic model. Using CCK-8, qRT-PCR, Western Blot, and gene overexpression techniques, the molecular mechanism was further explored.

**Results:**

The Rhodiola rosea treatment group showed significantly better improvements in DAS28, CRP, ESR, TNF-*α*, and IL-6 compared to the conventional treatment group (*p* < 0.05). Imaging assessment showed greater reductions in synovial thickness and blood-flow signals in the knee and metacarpophalangeal joints in the Rhodiola rosea group than in the conventional treatment group. The serum HIF-1α level in the Rhodiola rosea group was significantly lower, and the incidence of adverse reactions (18.92%) was significantly lower than that of the conventional treatment group (34.88%), without any serious adverse events. The *in vitro* experiments showed that Rhodiola rosea injection (RRI) reduced hypoxia-induced expression of HIF-1α and its downstream factors MMP-3, VEGF, COX-2, and ICAM-1.

**Conclusion:**

Rhodiola rosea was associated with improved disease activity and inflammatory markers in high-altitude RA patients, with a favorable safety profile. The *in vitro* findings suggest that inhibition of HIF-1α signaling may be one potential mechanism, although prospective and translational validation is still required.

## 1 Introduction

Rheumatoid arthritis (RA) is a chronic autoimmune disease characterized by synovial inflammation, joint destruction, and systemic complications (1–3). Its pathogenesis involves immune dysregulation and excessive pro-inflammatory cytokines (4, 5). Epidemiological studies show that in high-altitude regions, chronic hypoxic exposure exacerbates synovial inflammation, tissue damage, and therapeutic resistance (6, 7), highlighting the need for region-specific treatment strategies.

Hypoxia is a key driver of RA progression at high altitudes, primarily through activation of hypoxia-inducible factor-*α* (HIF-α), which promotes pro-inflammatory mediators and pathological angiogenesis (8). Targeting HIF-*α*-related pathways thus represents a promising therapeutic strategy. Rhodiola rosea, a Tibetan medicinal herb distributed in cold high-altitude regions, possesses anti-inflammatory, antioxidant, and anti-hypoxic properties (9–11). Its major bioactive constituents, salidroside and rosavin, have been shown to modulate inflammatory responses and alleviate hypoxia-induced tissue injury (9, 12, 13). Modern pharmacological studies have further demonstrated that Rhodiola rosea improves hypoxic tolerance and oxidative stress by regulating hypoxia-associated signaling pathways and suppressing inflammatory cytokine expression (14). However, despite its broad pharmacological activities under hypoxic conditions, the specific mechanisms underlying the therapeutic effects of Rhodiola rosea in RA remain largely unexplored. Moreover, emerging studies have demonstrated that salidroside, one of the major active constituent of Rhodiola rosea, exerts anti-inflammatory and protective effects in various hypoxia-related diseases through modulation of the HIF-1α signaling pathway. However, Rhodiola rosea extract contains multiple bioactive components (e.g., rosavin, tyrosol, and cinnamic alcohol derivatives), and whether its anti-rheumatic activity in high-altitude RA patients is exclusively attributable to salidroside or involves synergistic effects of multiple constituents remains to be determined.

Among the HIF-*α* family members, HIF-1α is recognized as a key isoform mediating inflammatory responses and tissue remodeling. Salidroside has been reported to exert multiple protective effects through modulation of the HIF-1α signaling pathway, including attenuation of osteoporosis in ovariectomized rats (15), inhibition of hypoxia-induced angiogenesis in human retinal microvascular endothelial cells (16), and regulation of macrophage pyroptosis to alleviate atherosclerosis (17). Nevertheless, whether salidroside can exert anti-inflammatory effects in RA under high-altitude conditions through regulation of the HIF-1α pathway, and whether it can effectively improve clinical outcomes in RA patients, remains to be systematically investigated and clinically validated.

A growing body of evidence indicates that the hypoxic microenvironment in the joint cavity is a critical driver of RA pathogenesis (8). In RA synovium, hypoxia-inducible factor-1α (HIF-1α) accumulates markedly, activates inflammatory signaling pathways, promotes pathological angiogenesis, and enhances synovial cell proliferation and cartilage destruction (18). Notably, HIF-1α is not merely a downstream consequence of hypoxia; it directly participates in disease progression by regulating key effectors such as RANKL, VEGF, MMP-3, and COX-2, and by promoting fibroblast-like synoviocyte activation via the cGAS-STING pathway (19). Under high-altitude conditions, chronic hypoxic exposure further exacerbates HIF-1α activation and inflammatory progression, making this pathway a particularly relevant therapeutic target for high-altitude RA patients.

Despite these properties, the clinical efficacy of Rhodiola rosea in high-altitude RA patients and its potential regulation of HIF-1α have not been systematically investigated. To address this gap, this study provides clinical evidence of Rhodiola rosea treatment in high-altitude RA patients and combines it with *in vitro* mechanistic validation. The primary aim was to assess its clinical efficacy, and the secondary aim was to determine whether its effects involve suppression of HIF-1α signaling in hypoxic synovial cells.

## 2 Materials and methods

### 2.1 Study population

This retrospective controlled study included 160 outpatients with rheumatoid arthritis (RA) from a high-altitude region. Patients were not randomized; instead, group allocation was determined by the actual treatment they received based on clinical practice and physician—patient shared decision-making. Specifically, patients who received Rhodiola rosea injection in addition to conventional therapy constituted the Rhodiola rosea treatment group (*n* = 74), whereas those who received conventional therapy alone constituted the conventional treatment group (*n* = 86).

The inclusion criteria were as follows: (1) fulfillment of the 2010 American College of Rheumatology (ACR)/European League Against Rheumatism (EULAR) classification criteria for RA; (2) permanent residence at an altitude of ≥2,500 m for at least 5 years; (3) age between 18 and 70 years; (4) disease duration of ≥6 months; (5) complete clinical and laboratory data.

The exclusion criteria were: (1) presence of other autoimmune diseases, such as systemic lupus erythematosus or ankylosing spondylitis; (2) severe cardiac, hepatic, or renal dysfunction, or active infectious diseases; (3) known allergy to Rhodiola rosea preparations or conventional antirheumatic medications; (4) pregnancy or lactation.

This study was approved by the Medical Scientific Research Ethics Committee of the Medical Faculty, Qinghai University (Approval No. SL202303-37), and the study was conducted in accordance with the principles of the Declaration of Helsinki.

Potential confounding variables including demographic characteristics, comorbidities, previous treatment history, baseline HIF-1α levels, and concomitant medications were collected and compared between groups to assess baseline comparability.

### 2.2 Study design and treatment protocols

Patients in the conventional treatment group received standard western medical therapy, including leflunomide tablets (Qilu Pharmaceutical Co., Ltd., National Drug Approval No. H20203195) administered orally at 20 mg once daily, and methotrexate tablets (Guoyao Yixin Pharmaceutical Co., Ltd., National Drug Approval No. H20253213) administered orally at 10 mg once weekly. The specific medication regimen will be determined by the rheumatologist in charge of the treatment.

Patients in the Rhodiola rosea group received Rhodiola rosea injection in addition to conventional therapy. Specifically, 10 mL of Rhodiola rosea injection (Tonghua Yusheng Pharmaceutical Co., Ltd., National Drug Approval No. Z20060362), with salidroside as the primary active component, was diluted in 250 mL of 5% sodium chloride injection (Zhejiang Tianrui Pharmaceutical Co., Ltd., National Drug Approval No. H33022241) and administered by intravenous infusion once daily. According to previously validated analytical methods (20, 21), the concentration of salidroside in the injection formulation of the same manufacturer is approximately 3.6–3.9 mg/mL. Therefore, each 10 mL dose contains approximately 36–39 mg of salidroside, corresponding to a final infusion concentration of approximately 0.14–0.16 mg/mL.

Vital signs and adverse reactions were closely monitored during infusion. All treatment regimens were managed by the same rheumatology specialist team to ensure consistency in dosage, treatment duration, and clinical management. Concomitant medications that could influence disease activity or inflammatory markers, including systemic corticosteroids and non-steroidal anti-inflammatory drugs (NSAIDs), were permitted when clinically indicated and were recorded throughout the treatment period. Corticosteroids were prescribed at the discretion of the treating rheumatologist for disease flare control, whereas NSAIDs were used on an as-needed basis for pain management. Any dose adjustment, treatment interruption, or drug withdrawal was documented. Treatment adherence was assessed by reviewing medication discontinuation, dose adjustments, and drug withdrawal records.

### 2.3 Data collection

In this study, comprehensive data were collected to evaluate the therapeutic efficacy and potential mechanisms of Rhodiola rosea in patients with rheumatoid arthritis (RA residing at high altitude). Data collection included demographic characteristics, clinical indicators, adverse event monitoring, and imaging assessments.

Baseline demographic information comprised age, sex, disease duration, body mass index (BMI), and smoking history. Clinical efficacy was evaluated using the Disease Activity Score in 28 joints (DAS28), an internationally recognized tool for assessing RA disease activity. The DAS28 score is calculated based on the number of tender and swollen joints (out of 28 joints), patient global health assessment, and inflammatory markers such as C-reactive protein (CRP) or erythrocyte sedimentation rate (ESR). Higher DAS28 scores indicate more severe disease activity.

Inflammatory biomarkers, including CRP, ESR, tumor necrosis factor-*α* (TNF-α), and interleukin-6 (IL-6), were measured using standardized laboratory procedures. Fasting venous blood samples (5 mL) were collected and centrifuged at 3500 r/min for 10 min to obtain serum. ESR was measured using the Westergren method, CRP levels were determined by immunoturbidimetric assay, and serum concentrations of TNF-*α* and IL-6 were quantified by enzyme-linked immunosorbent assay (ELISA).

Serum hypoxia-inducible factor-1α (HIF-1α) levels were measured by ELISA. Fasting venous blood samples (3 mL) were centrifuged at 3000 r/min for 10 min, and the supernatant was collected for analysis. Optical density was measured at 450 nm according to the manufacturer’s instructions.

Imaging assessment was performed using color Doppler ultrasonography to evaluate synovial thickness and synovial blood flow signals in the knee joints and metacarpophalangeal joints. Changes in synovial inflammation before and after treatment were compared.

DAS28 scores and inflammatory markers (CRP, ESR, TNF-*α*, and IL-6) were assessed at three time points: baseline, 1 month after treatment initiation, and 6 months after treatment. Serum HIF-1α levels were also measured at corresponding time points to analyze changes between the Rhodiola rosea treatment group and the conventional therapy group.

All adverse events, including gastrointestinal discomfort, skin rash, and abnormal liver function, were recorded throughout the treatment period. Data collection and processing were conducted in strict accordance with standardized protocols. All data were independently verified by two investigators to ensure accuracy and consistency. Laboratory procedures were performed following standard operating procedures (SOPs), and equipment calibration was conducted regularly to ensure measurement reliability. In addition, a double-data entry system was used for data management to minimize human error. These measures were used to improve data accuracy and completeness.

### 2.4 *In vitro* cell experiments

#### 2.4.1 Cell culture and drug treatment

Human fibroblast-like synoviocytes derived from rheumatoid arthritis patients (HFLS-RA) were obtained from the American Type Culture Collection (ATCC, Cat. No. IM-H439). Cells were cultured in Dulbecco’s Modified Eagle Medium (DMEM; Gibco, Cat. No. 11966025) supplemented with 10% fetal bovine serum (FBS; ExCell Bio, Cat. No. FSP500) and 1% penicillin–streptomycin (Beyotime, Cat. No. C0222), and maintained at 37 °C in a humidified incubator containing 5% CO₂ under normoxic conditions.

Rhodiola rosea injection (RRI) was provided by Tonghua Yusheng Pharmaceutical Co., Ltd. (Approval No. Z20060362). To simulate a high-altitude hypoxic environment, cells were placed in a tri-gas incubator (Thermo Scientific, HERAcell VIOS 250i) and exposed to hypoxic conditions consisting of 1% O₂, 5% CO₂, and 94% N₂ for 24 h.

#### 2.4.2 Cell viability assay

**Figure 1:**
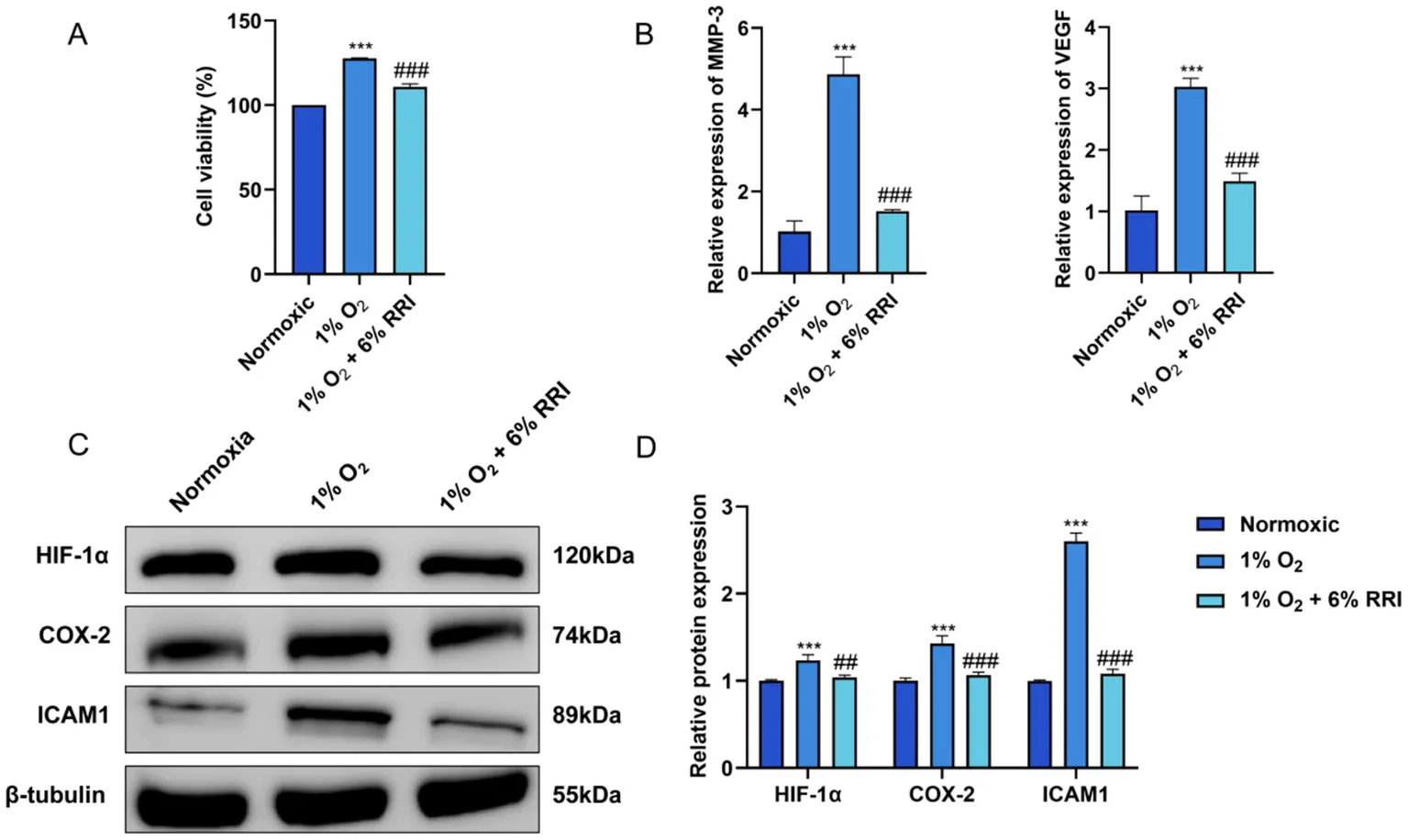
RRI inhibits hypoxia-induced HIF-1α pathway and inflammatory response. **(A)** CCK8 assay for cell viability; **(B)** qPCR detection of MMP-3 and VEGF mRNA levels; **(C)** Western Blot detection of HIF-1α, COX-2, ICAM1 protein levels; **(D)** Quantitative analysis of Western Blot results. ***p < 0.001 vs. Normoxia; ##*p* < 0.01, ###*p* < 0.001 vs. 1% O_2_.

**Figure 2:**
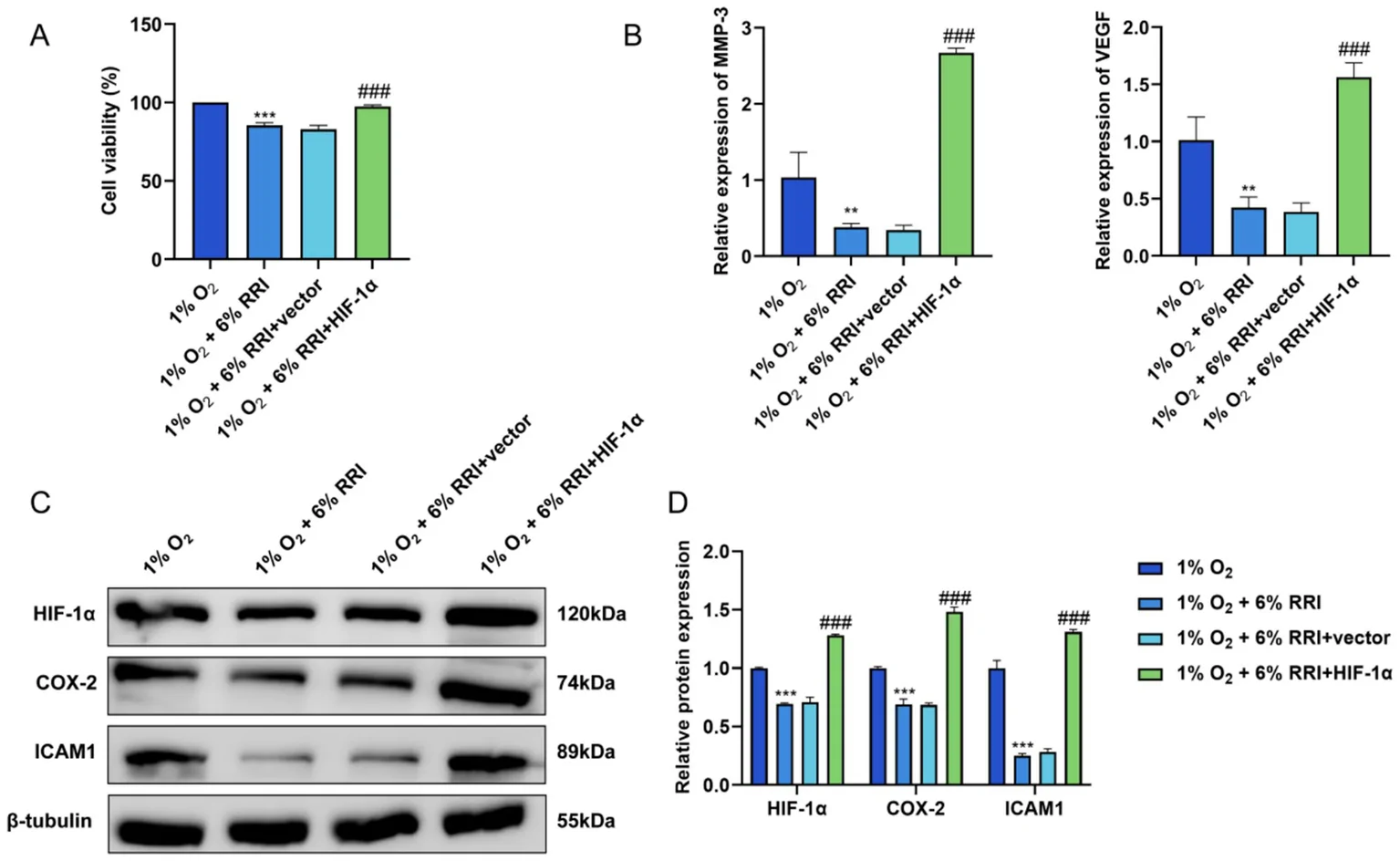
RRI exerts protective and anti-inflammatory effects by inhibiting the HIF-1α axis. **(A)** CCK8 assay for cell viability; **(B)** qPCR detection of MMP-3 and VEGF mRNA levels; **(C)** Western Blot detection of HIF-1α, COX-2, and ICAM1 protein levels; **(D)** Quantitative analysis of Western Blot results. **p < 0.01, ***p < 0.001 vs. 1% O_2_; ###*p* < 0.001 vs. 1% O_2_ + 6% RRI.

Cell viability was assessed using the Cell Counting Kit-8 (CCK-8) assay. HFLS-RA cells were seeded into 96-well plates at a density of 5 × 10^3^ cells per well and allowed to adhere for 24 h. The culture medium was then replaced with fresh medium containing different concentrations of RRI (0, 2, 4, 6, and 8%). Cells were subsequently incubated under normoxic or hypoxic conditions for an additional 24 h. Thereafter, 10 μL of CCK-8 solution (Beyotime, Cat. No. C0037) was added to each well and incubated for 2 h. Absorbance was measured at 450 nm using a microplate reader (BioTek, Synergy HTX). Cells were subsequently incubated under normoxic or hypoxic conditions for an additional 24 h. For experiments assessing the effect of RRI under hypoxia (Figure 1), a normoxic control without RRI was included as a baseline, but a normoxic control with RRI was not included because the primary comparison was between hypoxic vehicle and hypoxic RRI-treated cells. For HIF-1α overexpression experiments (Figure 2), all groups were maintained under hypoxia to focus on the interaction between RRI and HIF-1α, and a normoxic control was intentionally omitted as it would not contribute to testing the rescue hypothesis.

#### 2.4.3 Plasmid transfection and HIF-1α overexpression

To investigate the functional role of HIF-1α in the effects of RRI, a HIF-1α overexpression plasmid (HIF-1α group) and an empty vector plasmid (vector group) were constructed. Plasmids were transfected into HFLS-RA cells using Lipofectamine 3,000 transfection reagent (Invitrogen, Cat. No. L3000015) according to the manufacturer’s instructions. Forty-eight hours after transfection, the culture medium was replaced with medium containing 6% RRI, and cells were further incubated under hypoxic conditions (1% O₂) for 24 h before being harvested for subsequent analyses.

#### 2.4.4 Quantitative real-time PCR

Total RNA was extracted from HFLS-RA cells using TRIzol reagent (Invitrogen) according to the manufacturer’s protocol. Reverse transcription and quantitative PCR were performed using the HiScript II One Step RT-PCR Kit (Vazyme, Nanjing, China). Primer sequences are listed in Table 1, and GAPDH was used as the internal reference gene. Relative mRNA expression levels were calculated using the 2−^ΔΔCt^method.

**Table 1:** Primer sequences used for quantitative real-time PCR.

Gene name	Primer name	Primer sequence (5′–3′)
MMP-3	MMP-3-F	GACAAAGGATACAACAGGGACC
	MMP-3-R	TATCCAGCTCGTACCTCATTTC
VEGF	VEGF-F	TCTTCCAGGAGTACCCTGAT
	VEGF-R	TGTAGGAAGCTCATCTCTCC
GAPDH	GAPDH-F	AAGATCATCAGCAATGCCTCC
	GAPDH-R	AGGTTTTTCTAGACGGCAGG

MMP-3, matrix metalloproteinase-3; VEGF, vascular endothelial growth factor; GAPDH, glyceraldehyde-3-phosphate dehydrogenase.

#### 2.4.5 Western blot analysis

Total cellular proteins were extracted using RIPA lysis buffer (Beyotime, Cat. No. P0013B), and protein concentrations were determined using a bicinchoninic acid (BCA) protein assay kit (Beyotime, Cat. No. P0012). Equal amounts of protein were separated by SDS–polyacrylamide gel electrophoresis (SDS-PAGE) and subsequently transferred onto polyvinylidene fluoride (PVDF) membranes. Membranes were blocked with 5% non-fat milk (Beyotime, Cat. No. P0216) for 1 h at room temperature and then incubated overnight at 4 °C with the following primary antibodies: HIF-1α (Abcam, ab179483), COX-2 (Cell Signaling Technology, #12282), ICAM-1 (Cell Signaling Technology, #4915), and β-tubulin (Proteintech, 10,094-1-AP).

After washing, the membranes were incubated with a horseradish peroxidase (HRP)-conjugated secondary antibody (BIOSS, bs-0295G-HRP) for 2 h at room temperature. Protein bands were visualized using an enhanced chemiluminescence (ECL) detection reagent (Millipore, Cat. No. WBKLS0500) and captured using a chemiluminescence imaging system. Band intensities were quantified using ImageJ software.

### 2.5 Statistical analysis

All statistical analyses were performed using IBM SPSS Statistics software (version 23.0). For clinical data, continuous variables are presented as mean ± standard deviation (SD), while categorical variables are expressed as frequencies and percentages. Comparisons between two groups were conducted using the independent-samples t test; when data did not conform to a normal distribution, nonparametric tests (e.g., Mann–Whitney U test) were applied. Within-group longitudinal comparisons were analyzed using paired t tests.

For *in vitro* experiments, all assays were independently repeated at least three times, and data are presented as mean ± SD. Comparisons among multiple groups were performed using one-way analysis of variance (ANOVA), while comparisons between two groups were conducted using the *t*-test. A *p* value < 0.05 was considered statistically significant.

## 3 Results

### 3.1 Baseline characteristics

There were no statistically significant differences between the two groups in terms of average age, average disease duration, gender ratio, body mass index (BMI), and smoking history (all *p* > 0.05). Overall, the baseline characteristics of the two groups were comparable, providing a reliable basis for subsequent efficacy analysis (Table 2).

**Table 2:** Baseline characteristics and disease-related clinical features of patients.

Characteristic	Rhodiola treatment group (*n* = 74)	Conventional treatment group (*n* = 86)	t/χ^2^	*P* value
Age (years)	52.3 ± 8.1	51.7 ± 7.9	−0.493	0.622
Female, *n* (%)	54 (72.5)	60 (70.0)	0.074	0.786
Disease duration (years)	7.4 ± 3.3	7.7 ± 3.4	0.541	0.589
BMI (kg/m^2^)	23.8 ± 3.1	24.0 ± 3.4	0.386	0.7
Smoking history, *n* (%)	14 (18.8)	17 (20.0)	<0.001	>0.999
HIF-1α (ng/mL)	63.45 ± 16.76	60.03 ± 18.38	1.23	0.221
Hypertension, *n* (%)	17 (23.0)	25 (29.1)	0.76	0.382
Diabetes, *n* (%)	12 (16.2)	11 (12.8)	0.38	0.538
Angiocardiopathy, *n* (%)	8 (10.8)	5 (5.8)	1.33	0.249
Osteoporosis, *n* (%)	15 (20.3)	23 (26.7)	0.92	0.337
Interstitial lung disease, *n* (%)	10 (13.5)	10 (11.6)	0.13	0.719
Chronic gastric disease, *n* (%)	23 (31.1)	36 (41.9)	1.99	0.159
History of abnormal liver function, *n* (%)	12 (16.2)	11 (12.8)	0.38	0.538
Previous methotrexate treatment, *n* (%)	51 (68.9)	55 (64.0)	0.44	0.508
Previous leflunomide treatment, *n* (%)	32 (43.2)	35 (40.7)	0.11	0.745
Previous hydroxychloroquine treatment, *n* (%)	33 (44.6)	37 (43.0)	0.04	0.842
Previous NSAIDs treatment, *n* (%)	54 (73.0)	63 (73.3)	0	0.968
Previous glucocorticoid treatment, *n* (%)	24 (32.4)	39 (45.3)	2.78	0.095
Previous treatment with biologics/JAK inhibitors, *n* (%)	8 (10.8)	18 (20.9)	2.99	0.084
Previous treatment with traditional Chinese medicine/tibetan medicine, *n* (%)	21 (28.4)	26 (30.2)	0.07	0.797

Data are presented as mean ± standard deviation (SD) or *n* (%). Continuous variables were compared using independent-samples t tests and categorical variables using χ^2^ tests. BMI, body mass index; HIF-1α, hypoxia-inducible factor-1 alpha; NSAIDs, non-steroidal anti-inflammatory drugs; JAK, Janus kinase.

### 3.2 Between-group differences in therapeutic efficacy

Comparative analysis between the Rhodiola rosea treatment group and the conventional treatment group suggested that Rhodiola rosea was associated with significantly greater improvements in disease activity and systemic inflammation in patients with rheumatoid arthritis (RA) (Table 3). At baseline, no significant differences were observed between the two groups with respect to DAS28 scores, C-reactive protein (CRP), erythrocyte sedimentation rate (ESR), tumor necrosis factor-*α* (TNF-α), or interleukin-6 (IL-6) levels.

**Table 3:** Comparison of clinical efficacy between the Rhodiola rosea treatment group and the standard treatment group.

Indicator	Time point	Rhodiola group (*n* = 74)	Standard group (*n* = 86)	*t*	*P*
DAS28 score	Baseline	4.86 ± 0.64	4.91 ± 0.58	0.519	0.605
	1 month	3.21 ± 0.71	3.78 ± 0.65	5.299	<0.001
	6 months	2.89 ± 0.59	3.45 ± 0.63	5.771	<0.001
CRP (mg/L)	Baseline	12.3 ± 3.2	12.6 ± 3.0	0.611	0.542
	1 month	6.8 ± 2.4	9.1 ± 2.7	5.654	<0.001
	6 months	4.5 ± 1.8	7.3 ± 2.1	8.977	<0.001
ESR (mm/h)	Baseline	45.2 ± 10.8	46.1 ± 9.9	0.55	0.583
	1 month	31.6 ± 9.7	38.2 ± 10.1	4.197	<0.001
	6 months	22.7 ± 8.5	31.4 ± 9.2	6.177	<0.001
TNF-α (pg/mL)	Baseline	32.6 ± 5.4	33.1 ± 5.1	0.602	0.548
	1 month	25.8 ± 4.8	28.7 ± 4.9	3.768	<0.001
	6 months	18.3 ± 4.2	24.6 ± 4.5	9.105	<0.001
IL-6 (pg/mL)	Baseline	15.8 ± 3.7	16.2 ± 3.5	0.702	0.484
	1 month	11.9 ± 3.1	14.7 ± 3.4	5.409	<0.001
	6 months	8.7 ± 2.6	12.5 ± 3.0	8.492	<0.001
Knee synovial thickness (mm)	Baseline	5.2 ± 0.8	5.1 ± 0.9	0.744	0.458
	6 months	2.3 ± 0.7	3.8 ± 1.0	−11.103	<0.001
MCP synovial thickness (mm)	Baseline	3.6 ± 1.0	3.7 ± 1.1	−0.602	0.548
	6 months	1.9 ± 0.5	3.2 ± 0.8	−12.498	<0.001

Data are presented as mean ± SD. Between-group comparisons at each time point were performed using independent-samples *t*-tests. DAS28, Disease Activity Score in 28 joints; CRP, C-reactive protein; ESR, erythrocyte sedimentation rate; TNF-α: tumor necrosis factor-alpha; IL-6, interleukin-6; MCP, metacarpophalangeal joint.

After treatment, the patients in the Rhodiola group showed significantly greater improvement in disease activity and inflammatory biomarkers compared to those who only received conventional treatment. Significant differences were observed between the groups at both 1 month and 6 months (all *p* < 0.05) (Table 3).

As shown in Supplementary Table 1, there were no significant differences between the two groups regarding methotrexate or leflunomide usage, dosage, treatment duration, dose adjustment rates, medication interruption, or drug withdrawal (all *p* > 0.05). The proportion of patients who discontinued medication or withdrew from the study was also comparable between the two groups (both *p* > 0.05). Regarding other drugs that might affect disease activity, there was no significant difference in the use of systemic corticosteroids between the two groups during treatment (*p* > 0.05). In the Rhodiola treatment group, 3 patients occasionally used non-steroidal anti-inflammatory drugs (NSAIDs) to relieve pain, while in the Conventional treatment group, 8 patients did so. There was no statistically significant difference in the use of NSAIDs between the two groups (*p* > 0.05).

**Figure 3:**
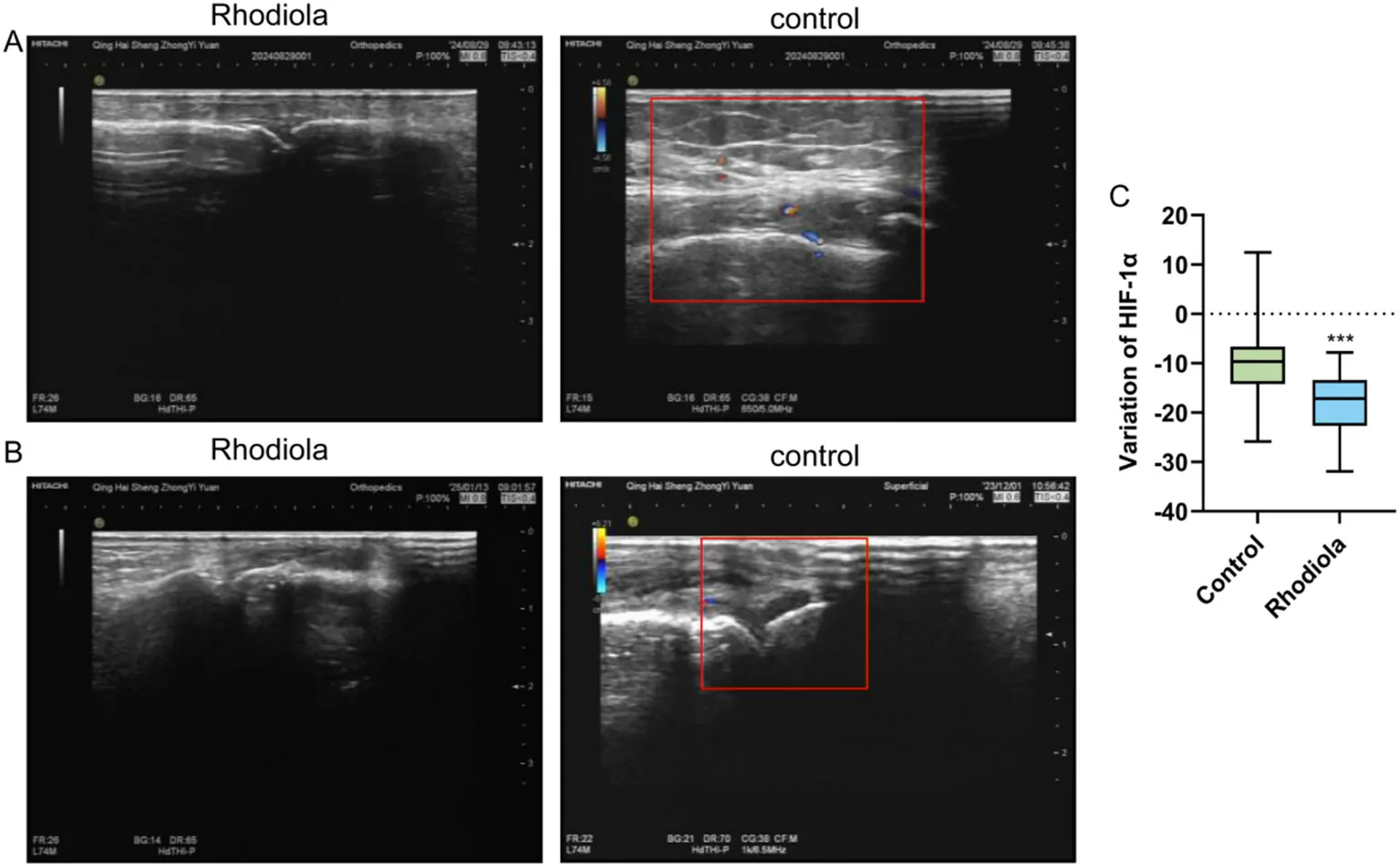
Imaging assessment and changes in HIF-1α. **(A)** Knee joint synovial imaging. **(B)** Metacarpophalangeal joint synovial imaging. **(C)** Changes in HIF-1α in the two groups of patients, ***p < 0.001 vs. Control.

Imaging assessment showed greater reductions in synovial thickness and synovial blood-flow signals in the Rhodiola rosea group than in the conventional treatment group (all *p* < 0.001) (Figures 3A,B). In addition, as shown in Figure 3C, the median change in serum hypoxia-inducible factor-1α (HIF-1α) levels was significantly greater in the Rhodiola rosea group than in the conventional treatment group (*p* < 0.001).

### 3.3 Analysis of adverse events

The incidence of adverse events was evaluated in the Rhodiola rosea treatment group (*n* = 74) and the conventional treatment group (*n* = 86). As shown in Table 4, the overall incidence of adverse events in the Rhodiola rosea group was 18.92%, which was significantly lower than that observed in the conventional treatment group (34.88%, *p* < 0.05).

**Table 4:** Adverse reaction monitoring.

Type of adverse reaction	Rhodiola treatment group (*n* = 74)	Conventional treatment group (*n* = 86)	χ^2^	*P* value
Overall adverse reactions	14 (18.92%)	30 (34.88%)	4.316	0.038
Gastrointestinal discomfort	8 (10.81%)	18 (20.93%)	2.3	0.13
Allergic reactions	6 (8.11%)	6 (6.98%)	<0.001	>0.999
Abnormal liver function	0 (0.00%)	5 (5.81%)	2.728	0.099
Dizziness or fatigue	0 (0.00%)	7 (8.14%)	4.504	0.034
Serious adverse events	0 (0.00%)	0 (0.00%)	0	0.343

Data are presented as *n* (%). Comparisons were performed using the χ^2^ test or Fisher’s exact test where appropriate. SAE, serious adverse event.

Specifically, no cases of dizziness or fatigue were reported in the Rhodiola rosea group, whereas the incidence in the conventional group was 8.14%, representing a statistically significant difference (*p* < 0.05). With respect to other adverse event categories, the incidence of gastrointestinal discomfort was lower in the Rhodiola rosea group than in the conventional group, while allergic reactions occurred slightly more frequently in the Rhodiola rosea group. Abnormal liver function was not observed in the Rhodiola rosea group but was reported in 5.81% of patients receiving conventional treatment. However, these differences did not reach statistical significance (all *p* > 0.05).

Importantly, no serious adverse events were observed in either group during the study period.

### 3.4 Effects of RRI on the viability of HFLS-RA cells

**Figure 4:**
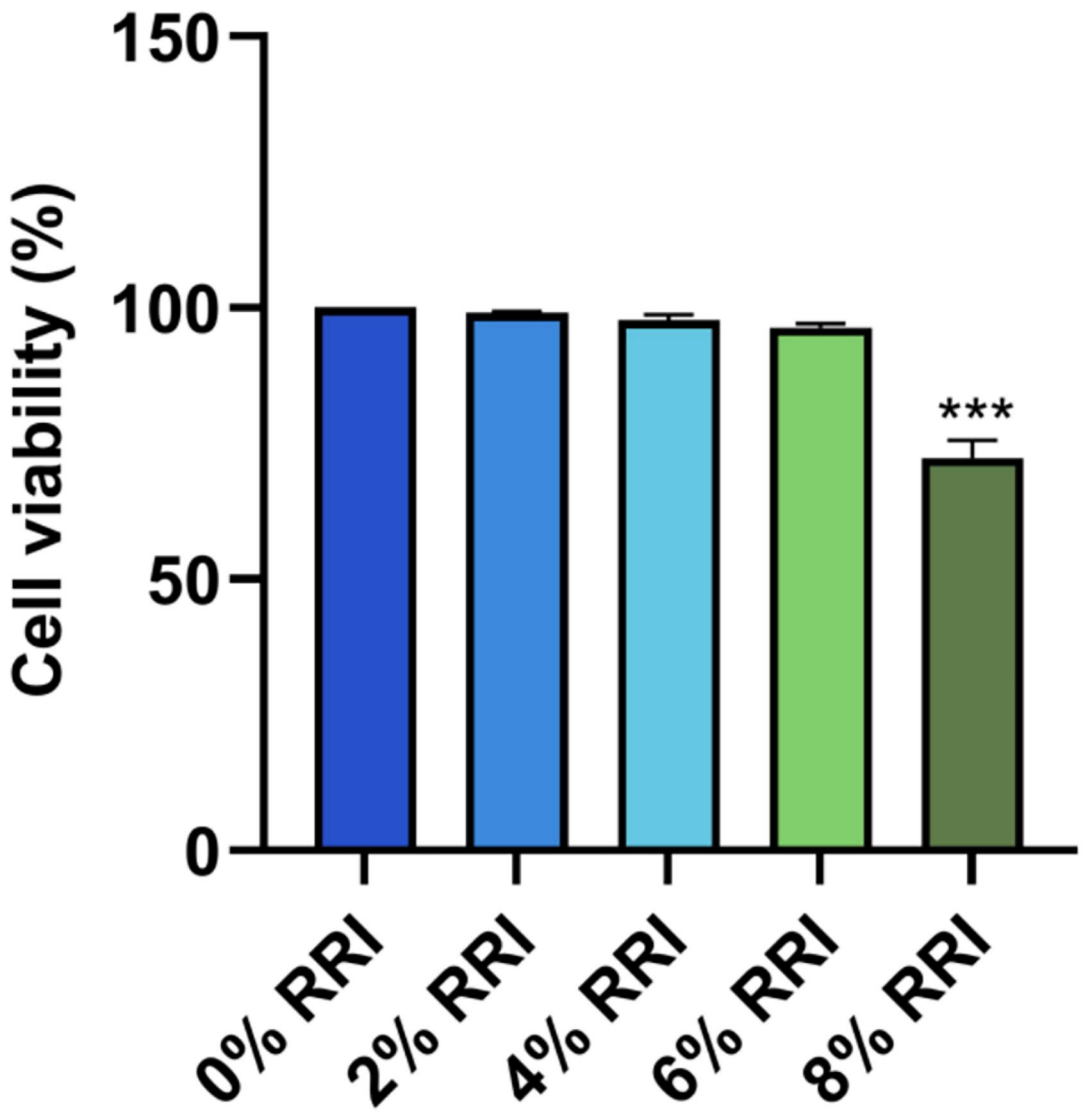
The effect of Rhodiola rosea injection on the viability of HFLS-RA cells. ***p < 0.001 vs. 0% RRI.

To evaluate the cytotoxicity and appropriate working concentration of RRI in fibroblast-like synoviocytes derived from patients with rheumatoid arthritis (HFLS-RA), cell viability was assessed using the CCK-8 assay following treatment with increasing concentrations of RRI (0–8%). As shown in Figure 4, no significant reduction in cell viability was observed in HFLS-RA cells treated with RRI at concentrations ranging from 2 to 6% compared with the 0% RRI group (*p* > 0.05). In contrast, exposure to 8% RRI resulted in a marked decrease in cell viability (*p* < 0.001). A concentration of 6% RRI was selected for subsequent experiments.

### 3.5 RRI suppresses hypoxia-induced HIF-1α signaling and inflammatory responses

To mimic the effects of high-altitude hypoxic conditions on rheumatoid arthritis (RA) synovium, HFLS-RA cells were cultured under hypoxic conditions (1% O₂). Compared with normoxic conditions, hypoxia significantly enhanced the viability of HFLS-RA cells. Treatment with 6% RRI significantly reversed the hypoxia-induced increase in cell viability (*p* < 0.001; Figure 1A).

To further elucidate the underlying molecular mechanisms, we examined the activation status of hypoxia-related signaling pathways. Hypoxic exposure robustly activated the HIF-1α signaling pathway and markedly upregulated the expression of downstream genes associated with joint destruction, including the matrix-degrading enzyme MMP-3 and the angiogenic factor VEGF at the mRNA level (*p* < 0.001; Figure 1B). In parallel, hypoxia significantly increased the protein expression of the inflammatory mediator COX-2 and the cell adhesion molecule ICAM-1 (*p* < 0.001; Figures 1C,D). Importantly, intervention with 6% RRI significantly attenuated hypoxia-induced activation of the HIF-1α pathway and concomitantly reduced the expression levels of MMP-3, VEGF, COX-2, and ICAM-1 (*p* < 0.01; Figures 1B,D).

### 3.6 RRI exerts protective and anti-inflammatory effects through inhibition of the HIF-1α axis

To directly determine whether HIF-1α serves as a critical molecular target mediating the effects of RRI, HIF-1α overexpression experiments were performed. Under hypoxic conditions, forced overexpression of HIF-1α significantly attenuated the inhibitory effect of 6% RRI on abnormal synoviocyte proliferation compared with the empty vector control (*p* < 0.001; Figure 2A). In parallel, HIF-1α overexpression partially reversed the suppressive effects of RRI on the mRNA expression levels of MMP-3 and VEGF (*p* < 0.001; Figure 2B).

Western blot analysis further demonstrated that HIF-1α overexpression markedly counteracted the RRI-induced downregulation of HIF-1α, COX-2, and ICAM1 protein expression (*p* < 0.001; Figures 2C,D).

## 4 Discussion

This study represents the first systematic evaluation of the clinical efficacy of Rhodiola rosea in patients with rheumatoid arthritis (RA) living in high-altitude regions and further explores its potential anti-inflammatory mechanism through regulation of the hypoxia-inducible factor-1*α* (HIF-1α) signaling pathway using *in vitro* experiments. The results demonstrated that patients in the Rhodiola treatment group exhibited significantly greater improvements in disease activity scores (DAS28), reductions in inflammatory cytokine levels, and reversal of synovial pathological changes compared with those receiving conventional therapy alone. Moreover, no serious adverse events were observed in the Rhodiola group, indicating favorable safety and tolerability.

The observed improvements in DAS28, CRP, ESR, TNF-*α*, and IL-6 were significantly greater in the Rhodiola rosea group than in the conventional treatment group. Previous studies have shown that Rhodiola extracts suppress the NF-κB signaling pathway and reduce pro-inflammatory cytokines in chronic inflammatory diseases (22, 23). Consistent with these mechanisms, the present study observed marked decreases in serum TNF-α and IL-6 levels in patients treated with Rhodiola, further supporting its immunomodulatory and anti-inflammatory role in RA (24, 25). However, most prior evidence on Rhodiola in arthritis has come from animal models under normoxic conditions. For example, a recent study demonstrated that Rhodiola rosea extract (RSE) alone significantly decreased arthritic parameters in the collagen-induced arthritis (CIA) model, and when combined with sub-therapeutic methotrexate, it further reduced inflammatory markers including IL-6, IL-17A, MMP-9, and CRP in an adjuvant arthritis (AA) model (26). The present study provides the first controlled evidence specifically in the high-altitude RA population, where chronic hypoxic exposure is known to exacerbate synovial inflammation and therapeutic resistance (6, 7). The greater efficacy observed here may be partly attributable to the anti-hypoxic properties of Rhodiola rosea, which are particularly relevant under high-altitude conditions.

Hypoxia is a critical driver of RA progression at high altitudes, largely through aberrant upregulation of HIF-1α, which activates inflammatory pathways and promotes tissue damage (27). Our clinical data showed that Rhodiola treatment was associated with lower serum HIF-1α levels. *In vitro*, hypoxic conditions (1% O₂) significantly enhanced HFLS-RA cell viability, consistent with the pathological proliferation and apoptosis resistance of RA synoviocytes. Treatment with RRI reversed this effect and suppressed the expression of HIF-1α protein as well as its downstream effectors MMP-3, VEGF, COX-2, and ICAM-1, which are involved in joint destruction, angiogenesis, and inflammation. Furthermore, HIF-1α overexpression experiments revealed that forced upregulation of HIF-1α significantly attenuated the protective effects of RRI, confirming that the anti-arthritic effects are at least partly dependent on inhibition of the HIF-1α signaling axis. The relationship between HIF-1α and NF-κB pathways under hypoxic conditions in RA remains incompletely understood. Hypoxia can activate both pathways (28), and while our data support that Rhodiola suppresses HIF-1α and its downstream effectors, previous studies have implicated NF-κB as another target. We cannot exclude the possibility that Rhodiola acts upstream of both or that HIF-1α modulates NF-κB activity. The present study does not resolve this hierarchy, and additional mechanistic studies are required to determine whether Rhodiola directly and independently inhibits NF-κB or whether the observed NF-κB suppression is secondary to HIF-1α inhibition.

The mechanistic link between Rhodiola rosea and HIF-1α inhibition is supported by several lines of published evidence. In cancer models, Rhodiola rosea extracts and salidroside inhibit the mTOR pathway and reduce angiogenesis through down-regulation of HIF-1α/HIF-2α expression (29). More recent studies have shown that salidroside directly binds to HIF-1α, suppressing HIF-1α-induced pyroptosis in macrophages (30). Salidroside also alleviates bone tissue hypoxia and decreases HIF-1α expression via downregulating RANKL, VEGF, IL-6, and ANGPTL4 in an osteolysis model (31). Nevertheless, direct evidence of Rhodiola rosea extract or salidroside specifically inhibiting HIF-1α in RA synovial cells remains limited, and the present study contributes to filling this gap.

It is important to note that this study utilized Rhodiola rosea injection (a multi-component extract) in both the clinical trial and *in vitro* experiments, rather than purified salidroside. Salidroside is recognized as the major bioactive constituent, and the injectable formulation used contains approximately 36–39 mg of salidroside per 10 mL dose. However, the extract contains multiple additional bioactive components—including rosavin, tyrosol, and cinnamic alcohol derivatives—that may independently or synergistically contribute to anti-inflammatory and anti-hypoxic effects. Experimental evidence has demonstrated that salidroside alone exerts anti-arthritic activity in vitro and in animal models. For example, salidroside suppresses the growth and inflammatory response of RA-FLSs by inhibiting the PI3K/AKT signaling pathway (32), and reshapes the Th17/Treg immune balance through regulation of the STAT3/HIF-1α/RORγt signaling axis (33). Furthermore, salidroside has been shown to inhibit HIF-1α signaling and alleviate bone tissue hypoxia in an LPS-induced osteolysis model (31). Nevertheless, whether salidroside fully recapitulates the anti-rheumatic efficacy of the whole extract formulation—particularly in the context of high-altitude RA—remains unknown. As noted by Li et al., both Rhodiola rosea extracts and salidroside can modulate HIF-1α expression, but they may exert opposing effects on cellular outcomes depending on physiological context (e.g., cancer vs. normal tissues) (29), raising the possibility that interactions among multiple constituents may be required for optimal therapeutic benefit under hypoxic conditions. Direct head-to-head comparisons of equimolar salidroside versus the original extract in hypoxic synovial cell models and *in vivo* arthritis models are therefore necessary. With respect to safety, the incidence of adverse events in the Rhodiola treatment group (18.92%) was significantly lower than that in the conventional therapy group (34.88%), and no cases of abnormal liver function were observed in the Rhodiola group. This contrasts with the potential hepatotoxicity associated with commonly used antirheumatic drugs such as methotrexate (34), underscoring the safety advantages of Rhodiola as a plant-derived therapeutic agent. The absence of liver function abnormalities also suggests that adding Rhodiola may not exacerbate, and could potentially mitigate, the hepatotoxic burden of conventional therapy, although this hepatoprotective effect was not directly tested in our patient cohort. Notably, comparable adherence, dose-adjustment rates, and concomitant medication use between groups reduce, but do not eliminate, the possibility that background therapy differences influenced the observed clinical outcomes.

Despite these encouraging findings, several limitations should be acknowledged. First, the retrospective, non-randomized design may have introduced selection bias and confounding by indication. No blinding was performed for patients, clinicians, outcome assessors, or data analysts, increasing the risk of performance and detection bias. Although measured baseline characteristics were generally comparable, residual confounding from unmeasured factors (e.g., patient preference, socioeconomic status, adherence behavior) cannot be excluded. Second, the relatively small sample size and single-region high-altitude population limit generalizability. Third, the mechanistic experiments were limited to one HFLS-RA cell model and were not validated in primary patient-derived synovial cells, immune-cell co-culture systems, or animal arthritis models. Fourth, the study did not include a direct comparative arm between Rhodiola rosea extract and purified salidroside, either in clinical or *in vitro* settings. Fifth, we did not perform mediation analysis to assess whether clinical improvement is statistically mediated by reduction in serum HIF-1α, nor did we obtain patient-matched synovial tissue or synovial fluid to verify that the HIF-1α suppression observed in cultured HFLS-RA cells also occurs in the joints of Rhodiola-treated patients. Consequently, the connection between clinical efficacy and the proposed HIF-1α mechanism remains correlative rather than causal. Future studies should incorporate prospective, randomized, placebo-controlled, assessor-blinded designs, larger sample sizes, multi-center recruitment, longer follow-up, and translational bridging analyses (e.g., synovial biopsies or imaging-guided molecular tracers) to establish a direct mechanistic link.

## 5 Conclusion

In summary, Rhodiola rosea extract formulation significantly improves disease activity and inflammatory responses in high-altitude RA patients with a favorable safety profile. *In vitro* experiments confirm that it attenuates hypoxia-induced synovial cell hyperproliferation and inflammatory activation by suppressing HIF-1α signaling and its downstream pathways. While the anti-rheumatic effects are likely mediated in part through HIF-1α signaling, further studies are required to determine whether salidroside alone or multiple bioactive components collectively contribute to the observed therapeutic benefits. These findings provide a potential adjunctive therapy for RA in hypoxic high-altitude environments and offer mechanistic support for developing natural compounds targeting hypoxia-associated inflammatory disorders.

## Data Availability

The original contributions presented in the study are included in the article/Supplementary material, further inquiries can be directed to the corresponding author.
