# Supplementary material for: The clinical efficacy and mechanism of Rhodiola rosea in treating rheumatoid arthritis in high-altitude areas by regulating the HIF-1α signaling pathway
Source: Front Med (Lausanne). 2026 Aug 7;13:1810329. doi: 10.3389/fmed.2026.1810329 (PMC13493261; doi:10.3389/fmed.2026.1810329)
Supplement: Supplementary file 1 [file Table_1.DOCX]

Supplementary Table 1. Comparison Table of Medication Information for the Two Groups of Patients During Treatment.

| Variable | Rhodiola treatment group (n = 74) | Conventional treatment group (n = 86) | t/χ² | *P* value |
| --- | --- | --- | --- | --- |
| Methotrexate treatment, n (%) | 51 (68.9) | 57 (66.3) | 0.13 | 0.722 |
| Methotrexate dosage, mg/week | 11.27 ± 2.42 | 11.14 ± 2.50 | 0.28 | 0.778 |
| Duration of methotrexate therapy, week | 22.60 ± 2.08 | 22.60 ± 1.48 | -0.02 | 0.988 |
| Leflunomide treatment, n (%) | 28 (37.8) | 36 (41.9) | 0.27 | 0.605 |
| Leflunomide dosage, mg/day | 17.86 ± 4.18 | 17.78 ± 4.22 | 0.08 | 0.940 |
| Duration of leflunomide therapy, week | 22.89 ± 1.74 | 22.34 ± 2.45 | 1.05 | 0.298 |
| Methotrexate dosage adjustment, n (%) | 4 (5.4) | 12 (14.0) | 3.23 | 0.072 |
| Leflunomide dosage adjustment, n (%) | 9 (12.2) | 5 (5.8) | 2.01 | 0.157 |
| Dose adjustment of Rhodiola rosea injection, n (%) | 2 (2.7) | 0 (0.0) | 2.35 | 0.125 |
| Any adjustment to the drug dosage, n (%) | 13 (17.6) | 15 (17.4) | 0 | 0.983 |
| Discontinue medication, n (%) | 12 (16.2) | 10 (11.6) | 0.71 | 0.401 |
| Drug withdrawal, n (%) | 4 (5.4) | 6 (7.0) | 0.17 | 0.682 |
| Corticosteroid treatment, n (%) | 5 (6.8) | 12 (14.0) | 2.17 | 0.141 |
| NSAIDs treatment, n (%) | 3 (4.1) | 8 (9.3) | 1.71 | 0.191 |

Note: Data are presented as mean ± SD or n (%). Continuous variables were analyzed using independent-samples t tests and categorical variables using χ² tests or Fisher’s exact tests where appropriate. NSAIDs: non-steroidal anti-inflammatory drugs.
